# Effective treatment with a tetrandrine/chloroquine combination for chloroquine-resistant falciparum malaria in *Aotus* monkeys

**DOI:** 10.1186/1475-2875-12-117

**Published:** 2013-04-02

**Authors:** Zuguang Ye, Knox Van Dyke, Richard N Rossan

**Affiliations:** 1 Department of Pharmacology, Institute of Chinese Materia Medica, Academy of Traditional Chinese Medicine, Beijing, 100700, China; 2 Department of Biochemistry, Robert C Byrd Health Sciences Center of West Virginia University, 1 Med Center Drive, Morgantown, WV 26506, USA; 3 Gorgas Memorial Laboratory, Panama, Panama; 4 Present address: Las Vegas, Nevada, 89147, USA

**Keywords:** Tetrandrine, Chloroquine, Aotus monkeys, Chloroquine-resistant falciparum malaria, Synergism

## Abstract

**Background:**

In vitro evidence indicates that tetrandrine (TT) can potentiate the action of chloroquine 40-fold against choloquine-resistant *Plasmodium falciparum*. The key question emanating from that study is “would tetrandine and chloroquine be highly effective in a live Aotus monkey model with chloroquine-resistant parasites”. This study was designed to closely mimic the pharmacological/anti-malarial activity in man.

**Methods:**

The Vietnam Smith/RE strain of *P. falciparum*, which is chloroquine-resistant was used in this study. Previous experimental procedures were followed. Panamanian owl monkeys (*Aotus*) were inoculated with 5×10^6^ erythrocytes parasitized with the CQ-resistant strain of *P. falciparum*. Oral drug treatment was with CQ (20 mg/kg) and/or tetrandrine at 15 mg/Kg, 30 mg/Kg or 60 mg/Kg or 25 mg/Kg depending on experimental conditions.

**Results and Discussion:**

Parasitaemia was cleared rapidly with CQ and TT while CQ treatment alone was ineffective. Recrudescence of malaria occurred after seven days post-infection. However, four animals were treated orally with TT and CQ parasites were cleared. It is likely that monkeys were cured via a combination of both drug and host immune responses. A single *Aotus* monkey infected with *P. falciparum* and untreated with drugs, died. No side effects were observed with these drug treatments.

**Conclusions:**

This combination of chloroquine and tetrandrine forms the basis of a new attack on chloroquine-resistant malaria - one based upon inhibition of the basis of chloroquine resistance, the multiple drug resistance pump. Previous studies demonstrated that the parasite MDR pump was found on parasite membranes using 3H azidopine photoaffinity labelling.

Since MDR-based choloroquine resistance is induced by chloroquine, the basis of the action of tetrandrine is the following: 1) tetrandrine inhibits the MDR pump by stimulating MDR ATPase which limits the energy of the pump by depletion of parasite ATP, 2) tetrandrine blocks the genetic factor which controls the induction of the pump. Therefore, it appears that the parasite cannot outsmart these mechanisms and produce a new mode of resistance. Only time will tell if this is correct.

## Background

Chloroquine has been, and probably still is, the most used anti-malarial drug throughout the world. The emergence and spread of chloroquine (CQ)-resistant falciparum malaria has evoked a search for new drugs effective against this drug-resistant parasite. However, the malarial parasite rapidly develops resistance to new anti-malarial drugs; for example, mefloquine and, more recently, artemisinin [1]. In 1987, Martin and co-workers explored a different method to attack the drug resistant parasite with the use of racemic verapamil combined with CQ. This combination reversed the resistance of the parasite *in vitro*[2], similar to its action in drug resistant cancer. However, at the extensive doses which reversed chloroquine resistance, verapamil exhibits cardiac toxicity in humans, which makes it impractical clinically.

Since then, much work has been focused on reversal of the resistance to CQ by using various compounds, such as calcium channel blockers [3] and their isomers [4-6], antidepressive drugs [7-9], antihistaminics [10-17]. However, most of the above combinations, which reversed the resistance *in vitro*, have not been successful *in vivo* either using the *Aotus* monkey [18] or doing clinical trials [19]. It has been previously reported that tetrandrine, a bisbenzyl isoquinoline, possesses a unique anti-malarial activity, especially selective against CQ-resistant *Plasmodium falciparum*[20], and that a combination of tetrandrine with CQ displayed a strong synergism against both CQ-sensitive and CQ-resistant *P. falciparum in vitro*[21]. The data obtained *in vitro* indicated that this TT/CQ combination was able to increase anti-malarial potency of chloroquine, such that a standard dose of CQ (1.5 g/patient) presumably might actually cure chloroquine-resistant falciparum malaria in humans.

Initially, an *in vitro* system was used to find active anti-malarial drugs or drug combinations. The situation *in vivo* is much more complex since biological activity is greatly affected by absorption (if given orally), distribution, metabolism, and excretion of the drug. Therefore, the data obtained *in vitro* cannot be directly extrapolated *in vivo*. Results with the tetrandrine-CQ combination *in vitro* must be tested in an *in vivo* system.

This study uses *Aotus* monkeys infected with a CQ-resistant strain of *P. falciparum* to confirm the effectiveness of the combination against CQ-resistant parasites *in vivo.* The aim of this study was to provide a pharmacodynamic basis for a possible human clinical trial with this combination.

## Methods

**Figure 1:**
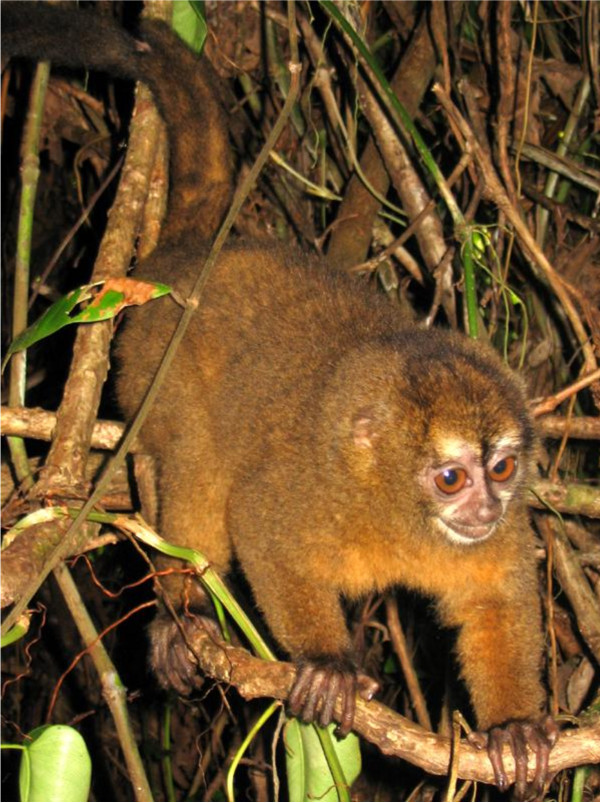
**The picture below is an Aotus monkey similar to the animals used in this study. **The original source was wikimedia commons and is used with permission.

The Vietnam Smith/RE strain of *P. falciparum*, which is CQ-resistant, was used in this study. Experimental procedures described previously were followed [18,22]. Briefly, Panamanian owl monkeys (*Aotus lemurinus lemurinus*)(Figure 1) were inoculated intravenously with 5 × 10^6^ erythrocytes parasitized with this CQ-resistant strain of *P. falciparum*. Five days after the inoculation, parasitaemia in the monkeys was about 5 × 10^3^/mm^3^, when the treatment with the combination initiated. In conducting the research described in this report, the investigators adhered to “Guide for the Care and Use of Laboratory Animals, prepared by the Committee on Care and Use of Laboratory Animals of the Institute of Laboratory Animals Resources of the Institute of Animal Resources, National Council ( DHEW publication No. (NIH) 78-23 Revised 1978).

A water-solution of both CQ diphosphate and tetrandrine dihydrochloride was prepared respectively at appropriate concentrations. Just before treatment, the two drug solutions were mixed and given orally by gastric intubation in a volume of 7.0 ml, followed by 7.0 ml water for rinse.

Treatment regimens in the experiments were divided into two categories. One-week therapy: After infection with the CQ-resistant parasite, the monkey was treated consecutively for seven days with CQ (20 mg/kg) combination with different doses of tetrandrine ranging from 15 to 60 mg/kg. If recrudescence occurred, the dose of tetrandrine in the combination was increased two times the initial dose for another one-week therapy. Two-week therapy: Instead of an increased dose of tetrandrine, the duration of treatment was changed from one week to two weeks.

A control, infected monkey was not given drug. In addition, five infected monkeys were administered CQ orally alone for seven consecutive days.

Blood smears were made daily during the experiment to monitor parasitaemia. After clearance of parasitaemia, a three-month follow-up was done to ascertain cure of infections.

## Results

Two experiments with *Aotus* monkeys treated with the combination of tetrandrine and CQ were conducted. In the first experiment, 15 mg/kg tetrandrine and 20 mg/kg CQ were given orally to three monkeys infected with CQ-resistant *P. falciparum* for seven consecutive days. In the second experiment, two monkeys were treated with the combination of 30 or 60 mg/kg tetrandrine and 20 mg/kg CQ for seven consecutive days. The results are summarized in Table 1.

**Table 1:** **Effect of the combination of tetrandrine and chloroquine against infected chloroquine-resistant *Plasmodium falciparum *malaria in *Aotus *monkeys**

**Expt. no.**	**Monkey no.**	**Dose**^**a**^** (Mg/kg)**	**Parasites cleared**	**Days from initial dose to parasite clearance**	**Days from final dose to recrudescence**	**Re-treating-post recrudescence (mg/kg)**	**Cured**
1	025	15(tt)	+			30 (tt) ^d^	(++)
		20(cq)		16	23	20 (cq)	
	055	15(tt)	+			30(tt) ^d^	(++)
		20(cq)		8	33	20(cq)	
	016	15(tt)	+			30 (tt) ^d^	(++)
		20(cq)		8	13	20 (cq)	
2	705	30(tt)	+			60 (tt) ^d^	(++)
		20(cq)		7	9	20 (cq)	
	706	60(tt)	+			120 (tt) ^d^	(++)
		20(cq)		7	14	20 (cq)	
	698^b^	-	-				
CQ^c^	520	20(cq)	-				
	432	20(cq)	-				
	349	20(cq)	-				
	544	20(cq)	-				
	631	20(cq)	-				

a tt and cq represent tetrandrine and chloroquine respectively.

b This monkey as a non-treated control died within 2 weeks after inoculation with the parasite.

c Monkeys were treated with CQ alone.

d Monkeys were cured after two week combinational cq and tt treatment=(++).

The CQ-resistant *P. falciparum* parasites were cleared in a mean of 9.2 days, ranging from 7 to 16 days after initial treatment with the drug combination (Table 1). Infections recrudesced in all the treated monkeys within one month after clearance of the parasite. In the first experiment, monkeys No. 025 and 016 were treated for another week with a higher dose of tetrandrine (30 mg/kg) combined with the CQ (20 mg/kg) after recrudescence occurred. One of the two monkeys was cured following the second treatment.

Following the recrudescence in the second experiment, re-treatment of monkeys No. 705 and 706 for one week with a higher dose of tetrandrine (60 mg/kg) did not cure the infections (recrudescence occurred again), although parasitaemia was rapidly cleared. A two-week therapy with a lower dose of tetrandrine (25 mg/kg) combined with the same dose of CQ (20 mg/kg) was used to treat monkeys No. 705, 706 and 725. No recrudescence occurred during the post-treatment examination period.

The infected monkey without any drug treatment died within two weeks. In monkeys treated with CQ alone, the parasite response to CQ alone was none or a slight suppression. The parasitaemia in three of five monkeys treated with CQ alone increased continuously during the treatment with CQ, while the parasitaemia in the remaining two monkeys was suppressed slightly.

## Discussion

The results obtained in *Aotus* monkeys infected with CQ-resistant *P. falciparum* showed that the tetrandrine combination with CQ can rapidly clear parasitaemia, while the parasite is not affected or only slightly affected by CQ alone (see Table 1). This is similar to a report that the Vietnam Smith/RE strain of *P. falciparum* is very resistant to CQ, because the drug exerted limited effects on parasitaemia in *Aotus* monkeys that received 20 mg/kg/day of CQ for several days [18]. Tetrandrine in combination with CQ may either increase total anti-malarial activity or inhibit the resistance of the parasite to CQ or both, so that a dose of CQ (20 mg/kg) in combination with tetrandrine exerts a remarkably potent anti-malarial effect against the CQ-resistant *P. falciparum* strain. Later work in cancer indicates that tetrandrine inhibits the transcription factor nuclear factor kappa b. This transcription factor is now known to control the stimulation of multiple drug resistance (MDR) mechanism. This cancer MDR mechanism is quite similar to the MDR pumping system that causes the exit pumping of chloroquine in drug resistant malarial parasites. Therefore, it should not be surprising that tetrandrine could inhibit either or both the production of the pump or the pumping action of the chloroquine- resistance pump (MDR). In fact, these events both happen in MDR drug resistant cancer cells. The tetrandrine/CQ combination creates a major synergism that was observed in the *in vitro* studies with chloroquine resistance in human falciparum malaria [21].

Recrudescence occurred after the one-week treatment with the drug combination. Following recrudescence in the first experiment, re-treatment of the monkeys with a larger dose of tetrandrine combined with chloroquine did clear the resistant malaria and cured infection in one monkey without the second treatment. In the second experiment, re-treatment with a higher dose of tetrandrine in the combination did not eliminate all the parasites in the monkeys, although the dose of tetrandrine in the combination was increased up to 120 mg/kg. Apparently, the increased dose of tetrandrine does not overcome the basis of recrudescence. Therefore, the length of therapeutic regimen was changed from one week to two weeks. No recrudescence occurred after the two-week treatment. It seemed that increasing the period of drug exposure to the parasite might be an effective method to overcome recrudescence. However, the likely possibility that host defense was partly responsible for the cure of these monkeys cannot be ruled out, because the monkeys that were treated with the two-week therapy are the same animals that had already received-the one-week treatment twice before; i.e., these monkeys had gone through a process of malarial infection: 7 days treatment - clearance of parasitaemia - recrudescence - 7 days retreatment - clearance of parasitaemia - recrudescence. In addition, it has been previously recognized there is some self-cure or spontaneous recovery that occurs naturally in these monkeys infected with *P. falciparum*. Maybe the cure of falciparum infection in these monkeys was elicited either by an effect of the two-week treatment, or partially by a spontaneous recovery, or more likely a combination of the drugs and this host defense. Therefore, it cannot be stated with certainty that the drugs were entirely responsible for the cure. Although the cause of recrudescence is not clear, the following possibilities were considered. Either the initial duration of exposure of the parasite to the drug combination is too short to kill all the parasites, or the concentration of tetrandrine is not enough for a long enough time at the site of action so that parasites that are hiding can survive, or both.

Tetrandrine is a bisbenzyl isoquinoline alkaloid, isolated from *Stephania tetrandra,* which has been used in traditional Chinese medicine either as an anti-rheumatic or analgesic agent. Tetrandrine is an approved drug in China, which has been used to treat hypertension and silicosis for centuries since it exerts known pharmacological activities on the cardiovascular system and produces anti-inflammatory activity [23-26]. Tetrandrine was found to exert little toxicity in clinical practice [23]. It can be given orally to humans at the dose of 200 mg or 300 mg/day for three months when used for treatment of silicosis [23]. Hence, tetrandrine is a relatively safe drug. Although no toxicological studies *per se* were performed on these monkeys, using the tetrandrine/CQ combination in this experiment; the monkeys appeared to easily tolerate orally a high dose of 120 mg/kg of tetrandrine in the combination with CQ at a dose of 20 mg/kg. One reason for other drug combinations failing when used at high doses in *in vivo* experiments, including clinical trials, was linked to their inherent toxicity. It seems that tetrandrine has relatively low toxicity. Therefore, it may be practical and helpful to conduct a clinical trial on the combination of tetrandrine and CQ on a small scale after further verifying the combinational safety of the tetrandrine with CQ.

## Conclusions

Based on data obtained in *Aotus* monkeys infected with CQ-resistant parasites, it can be concluded that the tetrandrine-CQ combination is effective against chloroquine-resistant *falciparum* malaria in *Aotus* monkeys. This combination can clear the parasitaemia rapidly, although some recrudescence occurred after treatment. In addition, a longer period of treatment; for example, a two-week’s application, might solve the recrudescence problem caused by the shorter treatment (one-week therapy). Artemisinin and its derivatives also displayed recrudescence in clinical trials as well as in animal experiments. By prolonging duration of the treatment with artemisinin or its derivatives, the recrudescence problem was essentially resolved. A further study of the effectiveness of a two-week treatment with the CQ/tetrandrine drug combination using a total of five *Aotus* monkeys to confirm the curative effectiveness of the two-week therapy is warranted.

There was not sufficient funds to continue these studies. Although statistical analysis was not accomplished, the cure of all five drug-treated animals after the 7–14 day combinational treatment leads one to an obvious conclusion that the combination of chloroquine and tetrandrine with an intact host immune system is an effective treatment of *Aotus*-infected falciparum malaria if treated for the correct amount of time. Both tetrandrine and chloroquine have been used in man as single entities for years. By combining the drugs to treat chloroquine resistant falciparum malaria in man would likely be an effective combination in man without major toxicity from either drug alone. Tetrandrine is metabolized in man and animals with great difficulty because it is a weak substrate for liver p-450 oxidation system. However, only a study in man with chloroquine-resistant, falciparum malaria could reveal the necessary data to confirm this research in *Aotus* monkeys. Since MDR-based choloroquine resistance is induced by chloroquine [27], the basis of the action of tetrandrine is the following: 1) tetrandrine inhibits the MDR pump by stimulating MDR ATPase which limits the energy of the pump by depletion of parasite ATP, 2) tetrandrine blocks the genetic factor which controls the induction of the pump. Therefore, it appears that the parasite cannot outsmart these mechanisms and produce a new mode of resistance. Only time will tell if this is correct.

## Competing interest

None of the three authors have a known conflict of interest with any material from this manuscript.

## Authors’ contribution

RNR performed the experimental/pharmacological work with *Aotus* monkeys at The Gorgas Memorial Laboratory in Panama. He was involved in both conception and design of this study. ZY was the first scientist to discover the effects of tetrandrine on the actual mechanism of chloroquine resistance. He developed the treatment schedule for this study and was involved with both conception and design of this study. The first effective *in vitro* screening system for anti-malarial drugs was developed in the laboratory of KVD for the United States Army during 1966–1970 during the Viet Nam war. KVD supplied the tetrandrine and chloroquine for the study and helped in study conception and experimental design. Both ZY and KVD wrote this manuscript. All authors read and approved the final manuscript.
